# The Clinical Value of Rodent Models in Understanding Preeclampsia Development and Progression

**DOI:** 10.1007/s11906-023-01233-9

**Published:** 2023-04-12

**Authors:** Sapna Ramdin, Sooraj Baijnath, Thajasvarie Naicker, Nalini Govender

**Affiliations:** 1 Department of Basic Medical Sciences, Faculty of Health Sciences, Durban University of Technology, Durban, South Africa; 2 Integrated Molecular Physiology Research Initiative, School of Physiology, Faculty of Health Sciences, University of the Witwatersrand, Johannesburg, South Africa; 3 Optics and Imaging Centre, Doris Duke Medical Research Institute, University of KwaZulu-Natal, Durban, South Africa

**Keywords:** Animal model, Blood pressure, Placenta, Preeclampsia, Pregnancy

## Abstract

**Purpose of Review:**

Preeclampsia (PE) is a leading global cause of maternal and fetal morbidity and mortality. The heterogeneity of this disorder contributes to its elusive etiology. Due to the ethical constraints surrounding human studies, animal models provide a suitable alternative for investigation into PE pathogenesis and novel therapeutic strategies. The purpose of this review is to compare and contrast the various rodent models used to study PE, in order to demonstrate their value in investigating and identifying different characteristics of this disorder.

**Recent Findings:**

Several approaches have been employed to create an appropriate animal model of PE, including surgical, genetic manipulation, and pharmacological methods in an attempt to mimic the maternal syndrome. Despite the absence of a model to completely model PE, these models have provided valuable information concerning various aspects of PE pathogenesis and novel therapeutic strategies and have led to the discovery of potential predictive markers of PE.

**Summary:**

Rodent and murine models have contributed significantly to the study of the pathology associated with specific aspects of the disorder. As a single fully encompassing animal model of PE remains absent, the use of a combination of models has potential value in understanding its etiology as well as in new treatment and management strategies.

## Introduction

Preeclampsia (PE) is a hypertensive disorder of pregnancy and a principal cause of maternal and fetal morbidity and mortality worldwide, resulting in approximately 46,000 maternal and 500,000 neonatal deaths per annum [1, 2]. Clinically, PE manifests as new-onset hypertension developing after 20 weeks of gestation (defined as blood pressure ≥ 140/90 mm Hg) together with one or more of the following: proteinuria (≥ 300 mg/day), maternal organ dysfunction, or uteroplacental dysfunction [3, 4••]. Thus far the only effective treatment is premature delivery and consequent early placental delivery, which is associated with the risk of neonatal morbidities [5].

The development of PE is a complex process that involves a number of dysfunctional physiological processes. A key event is abnormal placentation [6], characterized by reduced trophoblast invasion, inadequate remodeling of the maternal spiral arteries, and consequent placental ischemia [7]. This reduction in placental perfusion leads to placental hypoxia and oxidative stress [6]. The hypoxic placenta consequently secretes increased levels of anti-angiogenic markers, including soluble fms-like tyrosine kinase (sFlt-1) and soluble endoglin (sEng), into the maternal circulation, thereby inhibiting the bioavailability of proangiogenic vascular endothelial growth factor (VEGF) and placental growth factor (PlGF) [8, 9]. This angiogenic imbalance precedes the maternal syndrome of new-onset hypertension with or without the presence of proteinuria and with systemic endothelial dysfunction [10]. Preeclampsia is further categorized into early-onset PE, which can be diagnosed prior to 34 weeks of gestation and late-onset PE that is diagnosed from 34 weeks [11]. Early-onset PE is associated with abnormal placentation and fetal growth restriction, while late-onset PE is linked with maternal endothelial dysfunction [12].

While the precise etiology remains unclear, poor maternal and fetal outcomes continue to be exacerbated by inaccuracies in the identification and early diagnosis of women at high risk of PE development, especially in low- and middle-income countries. Therefore, identifying an appropriate animal model that mimics aspects of PE etiology will advance the current understanding of the conceptual framework underlying its development. However, since PE is a disorder of extreme heterogeneity, developing a gold standard model in this field remains challenging.

Current therapeutic interventions include anticoagulants such as aspirin, antihypertensive drugs such as labetalol, methyldopa, and nifedipine, as well as magnesium sulfate to prevent seizures [13]. Low-dose aspirin is reported to exert a prophylactic effect, by lowering the risk of early-onset PE development if administered before the 16th week of gestation [14]. However, it is ineffective in decreasing the risk of late-onset PE development [15]. Moreover, PE is identified by the American Heart Association, as a risk factor for impending circulatory disorders [16] and stroke [17]. Thus far, treatment strategies are only effective in managing the symptoms associated with PE and cannot be used to cure this disorder. With the absence of definitive treatment options and ethical limitations associated with the research in pregnancy, the development of animal models that cover the pathological aspects of PE is essential to increase our understanding of the disorder. Moreover, these models will enable the evaluation of novel treatment strategies in determining the safety and effectiveness of treatment interventions prior to clinical trial testing [18••].

The progress made towards understanding the development and progression of PE has seen a greater demand for the identification of novel agents for the treatment or prevention of PE [5]. Effective therapeutic interventions apart from delivery of the placenta would significantly improve maternal and neonatal health and pregnancy outcomes [5]. Despite the availability of extensive literature surrounding various models of PE development, this review will examine and provide a summary of the most extensively studied, as well as novel rodent models that have been used to study hypertension, proteinuria, maternal organ dysfunction, and fetal growth restriction in PE development.

## Animal Models of PE

Animal models to study PE development have been implemented using various methods. Preeclampsia may be induced via surgical and [19–21], pharmacological intervention [22, 23••], genetic, and immunological modification [24, 25••, 26, 27] and via the use of animals with pre-existing hypertension that develop superimposed PE [28, 29]. These models exhibit aspects of PE such as pregnancy-induced hypertension, elevated urinary protein levels, renal dysfunction, placental ischemia, and fetal growth restriction [18••]. However, the primary focus of several of these PE models lie in reproducing the maternal syndrome, and therefore, only a limited number of studies address initiating factors and primary stages of PE development [30].

Examining the initiation and progression of fetoplacental disorders in the first trimester in humans remains a challenge due to the potential danger associated with both maternal and fetal wellbeing [30]. Moreover, human clinical studies are associated with constraints that hinder a comprehensive investigation of the time-dependent processes that occur in PE development [31••]. In contrast, rodent models of PE support the study of the mechanisms that initiate development and progression of this disorder [23••, 25••, 32], since they have short gestations relative to humans. This permits the investigation of specific aspects of this multifactorial disorder, forming the preclinical basis for experimental testing and supporting the development of predictive tests and therapeutic strategies [6]. Moreover, in vivo, in vitro, and molecular methods may be explored to examine the underlying processes elicited by novel therapeutic interventions as well as effecting proof of concept experimental studies [18••, 31••].

Animal models have provided valuable insights into our knowledge of PE, including the mechanisms of deficient trophoblast migration [24, 33, 34] and placental ischemia [19, 35]. They have added to our understanding of endothelial dysfunction arising from the release of various factors from the hypoxic placenta [31••, 36]. Moreover, they have contributed significantly to the development of novel diagnostic strategies such as angiogenic screening platforms and immunoassays to aid in PE diagnosis [25••]. The use of Triage PlGF and the Elecsys immunoassay sFlt-1/PlGF ratio tests in conjunction with standard clinical assessment and subsequent clinical follow-up have been fully endorsed by the National Institute for Health and Care Excellence (NICE) [37]. Moreover, their clinical potential in helping to diagnose or rule out PE in women presenting with suspected symptoms between 20 and 34 weeks is undeniable. Albeit, the lack of sufficient evidence regarding their accuracy prevents their use as standard procedure for PE diagnosis [37].

Additionally, the accuracy of the BRAHMS Kryptor sFlt-1 and PlGF assays has been investigated, and the results have been found to be comparable to the Elecsys assays for sFlt1 and PlGF [38, 39•]. When used in combination with standard clinical methods of evaluation, the KRYPTOR assays have also displayed their utility in predicting the risk for PE-associated short-term adverse maternal and perinatal outcomes occurring within 2 weeks of presentation in women with suspected PE [38]. A recent study has demonstrated the KRYPTOR assays ability to rule in or rule out PE within a week and also proposes the clinical implementation of a simpler single decision sFlt1/PlGF ratio threshold of 66 as opposed to the currently used gestation-specific dual thresholds [39•].

## Rodent Models of PE

**Fig. 1:**
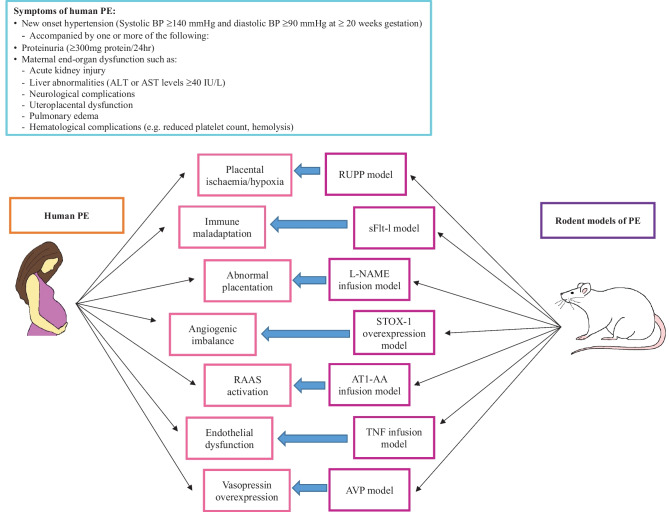
Various rodent models employed to study the pathogenic pathways associated with PE development

Rodent models have substantially contributed to the conceptual framework underlying the pathogenic mechanisms enabling a better understanding of the clinical manifestation of PE (Fig. 1). A brief overview of these models is displayed in Table 1. Since PE is a placental disorder, it is crucial for PE models to share common anatomical and functional features of human placentation [30]. Humans, rats, and mice are among the species that share hemochorial placentation. In rodents, placental development commences with the invasion of trophoblast cells into the maternal decidua which is followed by the remodeling of the maternal spiral arteries [40]. Humans and rodents share a similar profile of immune cells, including uterine natural killer cells in the maternal decidua [40]. While mice have enabled the study of placentation [41, 42], they exhibit shallow intrauterine trophoblast invasion, and placentation is superficial [43]. This is in direct contrast to human and rat placentation which is characterized by extensive trophoblast invasion and uterine arterial remodeling [44]. Furthermore, humans and rats share a similar discoid placental shape and deep invasiveness of the placenta but do differ in histological structure, as humans have a hemomonochorial structure, while rats display a hemotrichorial placenta [44, 45].

**Table 1:** A brief overview of current rodent and murine models of PE

**Model**	**Phenotypic induction**	**Potential mechanism**	**Species**	**Clinical features**	**References**
Reduced uterine perfusion pressure	Surgical	Placental ischemia	Rat	↑blood pressure, ↑urinary protein, ↑sFlt-1, ↑sEng, ↓PlGF, ↓VEGF, ↑ROS, ↑AT1-AA, ↑TNF-a, ↑regulatory T cells, ↑natural killer cells, ↑endothelin-1, ↓nitric oxide, ↓renal plasma flow, ↓GFR, ↓placental weight, fetal growth restriction	[19–21]
BPH/5	Spontaneous	Pre-existing hypertension	Mouse	↑blood pressure, ↑ urinary protein, ↑endothelial dysfunction, ↑glomerulosclerosis, ↓placental weight ↑fetal mortality, fetal growth restriction, ↓VEGF, ↓PlGF	[6, 29, 64]
Ad-sFlt-1 (vector)	Genetic	Angiogenic imbalance	Rat	↑blood pressure, ↑urinary protein, glomerular endotheliosis	[25••]
L-NAME	pharmacological	Endothelial dysfunction	Rat	↑blood pressure, ↑ urinary protein, ↑sFlt-1, ↓placental weight, fetal growth restriction	[22]
TNF-α	Pharmacological	Immune activation	Rat	↑blood pressure, ↑prepro-endothelin-1, ↓nitric oxide synthase, ↑AT1-AA	[93, 94]
Indoleamine 2,3-Dioxygenase knockout	Genetic	Immune activation	Mouse	↑blood pressure, ↑glomerular endotheliosis, fetal growth restriction, ↑urinary protein	[26, 27]
AT1-AA	Pharmacological	RAAS activation	Rat	↑blood pressure, ↑NK cells, ↑sFlt-1, ↑sEng, ↑prepro-ET-1, ↑AT1-AA	[50, 102, 104]
TLR3	Pharmacological	Immune activation	Rat	↑blood pressure, ↑systemic inflammation, ↑urinary protein, endothelial dysfunction	[105]
TLR7/8	Pharmacological	Immune activation	Mouse	↑blood pressure, endothelial dysfunction, splenomegaly, ↑placental inflammation	[108, 109]
TLR9	Pharmacological	Immune activation	Rat	↑blood pressure, ↑vasoconstriction, ↑vascular oxidative stress, ↑inflammation	[110]
STOX1	Genetic	Abnormal placentation	Mouse	↑systolic pressure, ↑ urinary protein, ↑renal capillary swelling, ↑sFlt-1, ↑sEng, fetal growth restriction, ↑cardiac hypertrophy, ↑renal artery resistance	[24, 33, 118, 119]
Dahl salt-sensitive rat	Spontaneous	Pre-existing hypertension	Rat	↑blood pressure, ↑ urinary protein, ↑sFlt-1, glomerulomegaly, fetal growth restriction	[28, 121]
Regulatory T cell depletion	Immunological	Immune activation	Mouse	blood pressure (no change), ↑TNF-a, ↑IFN, ↑IL-6, ↑IL-17, ↑MCP-1, ↑CXCL1, ↑fetal mortality	[130]
Arginine vasopressin	Pharmacological	Vasopressin (CNS)	Rat	↑blood pressure, ↑glomerular endotheliosis, ↑urinary protein, fetal growth restriction	[23••]

*Ad-sFlt-1 *adenovirus expressing soluble fms-like tyrosine kinase-1, *AT1-AA* angiotensin II type 1 receptor autoantibody, *BPH* blood pressure high, *CNS* central nervous system, *CXCL1* C-X-C motif chemokine ligand 1, *GFR* glomerular filtration rate, *IFN* interferon, *IL* interleukin, *L-NAME* L-NG-nitroarginine methyl ester, *MCP-1* macrophage chemoattractant protein-1, *NO* nitric oxide, *PlGF* placental growth factor, *ROS* reactive oxygen species, *sEng* soluble endoglin, *sFlt-1* soluble fms-like tyrosine kinase, *STOX1* storkhead box-1, *TLR* toll-like receptor, *TNF-a* tumor necrosis factor-a, *VEGF* vascular endothelial growth factor

Additionally, both mice and rats mimic human pregnancy-induced cardiovascular changes such as hypotension in early pregnancy, reduced pressor responses to angiotensin II, and decreased hematocrit, as well as elevations in cardiac output, stroke volume, and plasma volume [46, 47]. Thus, the extensive use of mice and rats to study hypertensive disorders of pregnancy such as PE is not surprising [45, 48]. Numerous experimental models of PE are also associated with elevated tissue levels of prepro-endothelin-1 mRNA. These models have been used to investigate whether the inhibition of the endothelin pathway could improve hypertension. Additionally, the reduced uterine perfusion pressure model, soluble fms-like tyrosine kinase rat model, BPH5 mouse model, and the nitric synthase inhibition (L-NAME) model have been explored as models to mimic PE development. The mean arterial pressure is reduced in the RUPP, sFlt-1 infusion, TNF infusion, and AT1-AA infusion models by the administration of the endothelin type A receptor antagonist [19, 49–51]. This implies that endothelin-1 is a potential common pathway in which placental factors exert their effects on the maternal vasculature to induce vasoconstriction and hypertension [31••]. These rodent models are also used to test vitamins (D and B) and drug (e.g., statins) interventions. Vitamin D administration in the RUPP model lowers blood pressure, endothelin-1, sFlt-1, and AT1-AA levels; however, fetal outcomes were not improved [52–54]. In contrast, the L-NAME model demonstrated reduced levels of sFlt-1 and TNF in response to vitamin D treatment [55].

### Established Rodent Models of PE

#### Reduced Uterine Perfusion Pressure Model

The reduced uterine artery perfusion (RUPP) rat model is the most widely characterized experimental model of placental ischemia. It reproduces the PE phenotype of endothelial dysfunction, glomerular endotheliosis, hemodynamic changes, and higher circulating levels of sFlt-1 and sEng [21]. This model is produced by clipping the aorta and uterine ovarian arteries on gestational day 14, resulting in a 40% decrease in uteroplacental perfusion along with a 20–30 mmHg increase in maternal mean arterial pressure on gestational day 19 in comparison to control groups [21]. The RUPP model has made substantial inroads in elucidating the role of the adaptive immune system in PE development [30]. Zenclussen and co-workers studied the role of inflammatory T cells in PE development and showed that the transference of activated T helper-1-like cells into healthy mice leads to the development of PE-like symptoms [56]. The RUPP model demonstrates elevated levels of inflammatory CD4 + T cells [57], along with reduced anti-inflammatory regulatory T cell levels [58]. This model also found that the adoptive transfer of CD 4 + T cells from RUPP rats to control rats induced hypertension, proteinuria, glomerular endotheliosis with concomitant elevated cytokines, and anti-angiogenic expression in circulation [59]. Albeit a major limitation of this model is its inability to replicate the immune mechanisms, deficient trophoblast invasion, and abnormal remodeling of the spiral arteries since the clipping of the lower abdominal aorta and uterine arteries is conducted mid-pregnancy (gestational day 14) [21]. Moreover, liver dysfunction and intrauterine growth restriction associated with human PE development are not replicated.

Moreover in the RUPP rat model, pravastatin treatment downregulates blood pressure and reactive oxygen species and improves angiogenic balance [60]; however, this was not observed in early human clinical trials [61, 62]. Pravastatin administration in early-onset PE women reduces the incidence of poor fetal outcomes but does not influence the concentration of plasma sFlt-1 levels or the sFlt-1:PlGF ratio in comparison to the placebo group [61, 62]. However, the focus still remains on angiogenic factors sFlt-1, PlGF, and VEGF due to the importance of angiogenic imbalance in PE pathogenesis. The administration of PlGF in RUPP rats has been reported to lower sFlt-1 levels, blood pressure, and proteinuria [63].

#### BPH/5 Mouse Model

This is a spontaneous or superimposed model of PE since an existing mild hypertension is present in the non-pregnant mice. Hypertension increases with pregnancy together with endothelial dysfunction, elevated uterine vascular resistance, placental dysfunction, and reduced litter size [29, 64]. This model was created through the continued mating of inbred BPH (blood pressure high)/2 mice leading to the development of the BPH/5 mouse strain [65]. A study using the BPH/5 model found that the maternal phenotype of PE may be initiated by the increased decidual expression of cyclooxygenase-2 (COX-2) and interleukin-15 [6]. This model has also been used to determine the effectiveness of therapeutic intervention of proangiogenic factor, where adenoviral delivery of VEGF_121_ inhibited the development of superimposed PE [66]. These findings highlight the therapeutic potential of early proangiogenic intervention in pregnancy-associated hypertensive disorders. Despite the pre-existing hypertension displayed by non-pregnant mice, the usefulness of this model in providing information of how pre-existing hypertension influences the pathogenesis of superimposed PE is valuable [30].

#### sFlt-1 Sprague Dawley Rat Model

The clinical importance of the sFlt-1/PlGF ratio encouraged research that evaluated its clinical value in the diagnosis and prediction of PE. This ratio has contributed substantially to the creation of automated angiogenic biomarker platforms (sFlt-1 and PlGF) to aid the diagnosis and prognosis of PE in high-income countries. Maynard and co-workers developed the novel experimental rat model which replicated clinical characteristics of PE [25••]. Their findings indicate that exogenous administration of sFlt-1 to pregnant rats produces hypertension, proteinuria, and glomerular endotheliosis, which are characteristic of PE [25••]. In contrast Thadhani and co-workers reported that sFlt-1 clearance by apheresis improve angiogenic balance in preeclamptic women and demonstrated lowered mean arterial pressure and extended gestation by up to 15 days [67, 68].

Maynard’s breakthrough study thus endorsed the development of the immunoassays, Alere Triage PlGF test and the Elecsys sFlt-1/PlGF from Roche, which have the potential to exclude PE diagnosis in women with suspected PE between 20 and 34 weeks [69]. The BRAHMS Kryptor sFlt-1 and PlGF assays have also demonstrated comparable accuracy to the Elecsys assays for sFlt1 and PlGF when ruling in or ruling out PE [38, 39•].

Additionally, this model was used to study the long-term effects of pregnancy-induced hypertension on maternal and fetal outcomes. The offspring of sFlt-1 treated mice exhibited elevated blood pressure levels and also reported that baseline maternal cardiovascular function was not adversely affected postpartum [70]. Off note, a shortcoming of this model is its inability to reproduce the liver dysfunction and intrauterine growth restriction associated with human PE development.

#### Nitric Oxide Synthase Inhibition (L-NAME)

The nitro-L-arginine methyl ester (L-NAME) model is a model of endothelial dysfunction associated with PE development. In PE, the nitric oxide pathway is defective and polymorphisms in nitric oxide synthase (NOS) exist [71, 72]. Using a rodent model, the administration of L-NAME inhibits NOS and produces PE-like characteristics such as high blood pressure, proteinuria, decreased glomerular filtration rate, and intrauterine growth restriction [73–76]. Additionally, early-onset PE (EOPE) and late-onset PE (LOPE) phenotypes may also be produced by altering the timing of L-NAME administration in pregnant Sprague Dawley rats [32]. The L-NAME rat and mouse models of PE have been utilized in testing potential treatment and biomarker predictor tests for PE development. Administration of sildenafil improves hypertension, proteinuria, and fetal outcomes in early- and late-onset cases of PE [36, 74, 77, 78] and downregulates plasma sFlt-1 and sEng levels [22]. Furthermore, urinary nephrin and podocin mRNA levels are significantly higher in both EOPE and LOPE L-NAME-treated rats in comparison to pregnant control rats, suggestive of the presence of podocyturia. A significant reduction in urinary mRNA levels of podocin in EOPE rats and nephrin levels in LOPE rats was demonstrated following treatment with sildenafil citrate [79].

In contrast, sildenafil treatment administered late in gestation to rats and pregnant women with PE is associated with poor outcome [80, 81]. The uncertainty of NOS regulation in PE development raises concerns regarding the validity of the L-NAME model in PE investigations [18••, 30, 65, 80, 82, 83•]. Despite the polymorphism in the NOS gene displayed in pregnant women with severe PE [71, 72], inconsistencies are reported in studies involving the genetic deletion of NOS in pregnant mice [84–86]. Reduced blood pressure has been reported in pregnant NOS knockout mice [85], in contrast to higher blood pressure observed in non-pregnant NOS knockout mice [84]. Non-pregnant mice administered with L-NAME also show elevations in blood pressure with aortic vascular contraction, suggestive that the PE-like symptoms produced by this model may not be pregnancy-specific [86].

#### Tumor Necrosis Factor Alpha Model

Tumor necrosis factor alpha (TNF) is a pro-inflammatory cytokine involved in physiological processes such as cellular proliferation and differentiation, apoptosis, cell proliferation, differentiation, apoptosis, and inflammation [87]. Higher levels of TNF are reported in preeclamptic compared to normotensive pregnancies and hypertensive pregnancies uncomplicated by PE [88–91]. Women with pregnancies complicated by early-onset PE have significantly elevated levels of serum TNF in contrast to women with late-onset PE [92]. Normotensive pregnant rats administered with TNF (50 ng/d) between gestational days 14–19 develop hypertension and express elevated levels of renal, placental, and aortic prepro-endothelin-1 [93]. The administration of an endothelin type A receptor antagonist in pregnant rats ameliorates hypertension induced by TNF [51]. Additionally this model proposes that TNF-induced hypertension may emanate from the decline in renal NOS expression [93] and increase in AT1-AA production [94]. A major limitation observed in this model is that the PE phenotypes produced in response to chronic administration of TNF may be an exaggeration of the pro-inflammatory state of pregnancy [83•].

#### Indoleamine 2,3-Dioxygenase Knockout Model

Indoleamine 2,3-dioxygenase (IDO) is a cytosolic hemeprotein which catalyzes the rate-limiting step in tryptophan breakdown [83•] and is essential in the T cell-mediated immune response [95]. The IDO mouse model is produced via IDO inhibition using 1-methyl-tryptophan [27]. These mice develop high blood pressure or proteinuria, and their placentae express edematous changes and fibrin deposits, and there is no fetal growth restriction [27]. Santillan and co-workers reported glomerular endotheliosis, intrauterine growth restriction, and proteinuria in IDO-knockout mice; no changes in placental morphology and blood pressure were observed [26]. Preeclamptic women display downregulated placental levels of IDO [96–98]; interestingly, this is not observed in placentae from pregnancies with fetal growth restriction but without hypertension [97–99]. Late-onset PE patients demonstrate significantly lower IDO expression on endothelial cells in comparison to women with early-onset PE [100]. A shortfall of this model is its inability to fully mimic the clinical characteristics of hypertension and placental abnormalities associated with human PE development; however, it supports investigations surrounding placental inadequacy and renal dysfunction in pregnancy.

#### Angiotensin II Type 1 Receptor Autoantibody Model

The angiotensin II type 1 receptor autoantibody (AT1-AA) mouse model was generated by administering AT1-AAs obtained from preeclamptic women into mice on day 13 of gestation [101]. These mice displayed significantly higher blood pressure, proteinuria, glomerular endotheliosis, elevated sFlt-1 and sEng levels, smaller placentae, and fetal growth restriction compared to the control group [101]. Normal pregnancy and PE are significantly affected by the renin-angiotensin system (RAS). Angiotensin II type 1 receptor autoantibodies (AT1-AA) are reported to be higher in some women with PE and are linked to other disorders such as systemic sclerosis, tissue fibrosis, hypertension, and reno-vascular disease [102]. These autoantibodies influence vasoconstriction and increase blood pressure through the stimulation of angiotensin II type 1 (AT1) receptors with transduction of signals via the MAPK/ERK pathway [102, 103]. LaMarca and co-workers have reported that pregnant rats infused with purified AT1-AA between gestational days 12–19 demonstrated high blood pressure and high serum AT1-AA levels as well as dysregulated angiogenic factor levels and increased tissue levels of prepro-endothelin-1 in comparison to normotensive pregnant control rats [50]. The endothelin system may play a role in the elevation of blood pressure induced by AT1-AA administration; this theory is corroborated by the report of endothelin type A receptors inhibiting the blood pressure response in AT1-AA-infused rats [104]. These studies display a significant interaction between inflammatory and angiogenic markers that are released in response to placental ischemia. A disadvantage of the AT1-AA mouse model is that some aspects observed are not specific to pregnancy. The study by Zhou and co-workers also assessed the involvement of AT1-AAs independently of excess sFlt1; they reported elevations in blood pressure in non-pregnant mice following treatment with IgG isolated from preeclamptic women [101]. In these animals, renal injury and elevations in urinary protein and sFlt-1 levels were absent, suggesting that AT1-AA in non-pregnant women would chronically induce high blood pressure in an autoimmune manner. Furthermore, in the case of pregnancy, PE symptoms may not be resolved post-delivery [83•].

#### Toll-Like Receptor Rat Model

The Toll-like receptor (TLR) rat model was induced by the activation of TLR3 in pregnant rats with a viral mimetic called polyinosinic:polycytidylic acid [105]. This results in elevated maternal blood pressure, systemic inflammation, proteinuria, and endothelial dysfunction [105]. This study was the first to demonstrate that activation of TLR signaling during pregnancy adversely effects maternal cardiovascular function. These receptors are a class of pattern recognition receptors which induce signaling cascades to elicit appropriate inflammatory responses to pathogen- and damage-associated molecular patterns [106]. These receptors are present at the maternal–fetal interface and function to ensure a successful pregnancy outcome; conversely, excessive TLR signaling may induce maternal systemic inflammation with adverse pregnancy outcomes [107]. This rat model has since been replicated in pregnant mice [108, 109], and mice treated with synthetic ligands for TLR7/8 have produced similar outcomes as earlier reports [109]. Low-dose of unmethylated CpG DNA administered to female rats have been shown to stimulate TLR9 signaling in late gestation and increases blood pressure, vasoconstriction, vascular oxidative stress, and inflammation [110]. Earlier studies in mice showed that activation of TLR9 with fetal DNA or high doses of CpG DNA resulted in poor fetal outcome, including increased fetal resorption and malformations [111–113]. The findings of these studies imply that the TLR-induced initiation of the innate immune system plays a role in the development of hypertension in pregnancy.

#### STOX1 Mouse Model

Storkhead box-1 (STOX1) is a transcription factor found in column extravillous trophoblast cell populations and facilitates cytotrophoblast invasion during normal placentation by regulating α-T-catenin expression [114, 115]. Alterations in STOX1 expression have been implicated in PE development [116, 117]. Overexpression of STOX1 in pregnant mice elevates systolic blood pressure in early pregnancy, proteinuria, occlusion of renal capillaries, fibrin deposition, and increases sFlt-1 and sEng expression [24, 33]. The increase in systolic blood pressure precedes placental development and indicates that abnormal placental development may not be responsible for the hypertension observed in this model. This finding also suggests that the placenta may not be the initiating organ of PE development [83•]. This model also reports increased renal artery resistance, cardiac hypertrophy, fetal growth restriction, and greater umbilical resistance [118, 119] and demonstrates left ventricular hypertrophy, cardiac fibrosis, and markers of inflammation and cellular stress up to 8 months postpartum [120]. A benefit of this model is its ability to replicate the early pathogenic aspects of PE as well as the later systemic features. However, a limitation of the STOX1 mouse model is the observation of increase in blood pressure in early gestation and non-comparable to what is observed in preeclampsia. Therefore, the hypertension observed cannot be attributed to abnormal placentation.

#### Dahl Salt-Sensitive Rat Model

The Dahl salt-sensitive rat has pre-existing hypertension and during the course of gestation develops further PE-like symptoms such as increase in blood pressure, proteinuria, glomerulomegaly, placental hypoxia, fetal growth restriction, and higher circulating levels of TNF and sFlt-1 [28]. This model represents a spontaneous or superimposed rat model of PE. Gillis and co-workers used this model to validate the usefulness of sildenafil citrate treatment in PE [121]. Their data confirm that administration of sildenafil citrate between gestational day 10 and 20 alleviated further increase in blood pressure and proteinuria, decreased uterine artery resistance, and aided fetal growth [121]. Nonetheless, a disadvantage of this model is the pre-existing hypertension exhibited by pregnant rats [30].

#### Arginine Vasopressin Mouse Model

Hypertension induced by arginine vasopressin (AVP) is characterized by low circulating renin-angiotensin system activity, which is also found in PE compared to normotensive pregnant women [23••]. AVP exerts its physiological functions via V1a and V2 receptors [122]; the activation of these receptors has been implicated in proteinuria, renal glomerular endotheliosis, and intrauterine growth restriction, respectively. Additionally, the V1b receptor is a regulator of adrenocorticotropic hormone secretion, which can exert its effects on the immune system and blood pressure [123]; cullin-5 plays a role in angiogenesis [124], while the oxytocin receptor is involved in pregnancy and labor [125].

Studies by Santillan and co-workers highlight the role of AVP in PE development; however, chronic infusion of AVP does not reproduce placental hypoxia in mice which is a characteristic of human PE [122]. This model demonstrates the potential of AVP as both a predictive biomarker for PE development as well as an initiator of this disorder. Plasma copeptin levels, a biomarker of AVP, was reported to be significantly higher at 6 weeks of gestation in PE cases compared to normotensive pregnancy [23••]. The chronic administration of AVP in pregnant mice replicated pregnancy-specific hypertension, glomerular endotheliosis, proteinuria, and intrauterine growth restriction, thus supporting the role of AVP in PE progression and copeptin as an early biomarker for PE prediction [23••].

Santillan and co-workers have further expanded this work, in that AVP infusion into pregnant C57BL/6 J mice reduced the levels of placental expression of placental growth factor, altered placental morphology, placental oxidative stress, and placental gene expression consistent with the characteristic features of human PE [122]. They have also demonstrated that AVP infusion throughout gestation in mice promoted pro-inflammatory T_H_1-associated interferon gamma in maternal plasma [126]. The effectiveness of AVP in inducing PE-like symptoms in a mouse model was therefore successfully demonstrated. This model proposes the concept that regulators of blood pressure are activated in the early stages of pregnancy,and could therefore be a potential new model for studying the origins of PE [127].

In addition, our laboratory has extrapolated the AVP mouse model of Santillan and co-workers to a Sprague Dawley rat model [128••]. Our findings demonstrate that chronic AVP infusion (150 ng/h) in pregnant rats over 18 days successfully reproduced the PE phenotype of elevated blood pressure (≥ 140/90 mmHg) [10], increased urinary protein levels, and fetal growth restriction. Albeit, our study models a mild case of PE development, and future studies should explore higher AVP dosages to induce more severe features of PE development. Additionally, our study did not confirm the levels of angiogenic markers such as PlGF and sFlt-1 which are commonly associated with PE.

Biochemical analysis revealed significantly upregulated serum alanine transaminase and triglyceride levels along with downregulated high-density lipoprotein levels in pregnant AVP-treated rats. We further demonstrate alterations in kidney morphology including a mild increase in mesangium, mild glomerular crescents, and reduced Bowman’s space in AVP-treated rats. An earlier study performed by our lab assessed liver injury in the AVP rat model and found that serum expression of the liver injury enzymes arginase and 5′-nucleotidase, as well as transforming growth factor-2, was significantly higher in pregnant rats treated with AVP [129••]. Our findings are indicative of acute pregnancy-initiated liver dysfunction and support the utility of this model in the study of PE development.

The novel AVP mouse model highlights the potential use of AVP as a predictive biomarker for PE development. This model recapitulates phenotypes consistent with human PE, most notably pregnancy-specific hypertension. The activation of V1a and V2 receptors has been implicated in proteinuria and renal glomerular endotheliosis and intrauterine growth restriction, respectively, in this model. Future studies using the AVP model should explore the roles of the V1b, the oxytocin receptor, and cullin-5 (VACM-1) in the pathogenesis of this disorder.

## Conclusion

Despite the advances made in understanding PE development, it continues to be a leading cause of maternal and fetal mortality and morbidity worldwide. This review provides a brief overview of various rodent and murine models that mimic PE development while also highlighting the associated limitations. We report that there is no ideal rodent model to date that fully epitomizes the phenotype of PE such as abnormal placentation, fetal growth restriction, pregnancy-specific hypertension, proteinuria, endothelial dysfunction, and an imbalance in angiogenic factors. Despite the invaluable contribution of the different models, they do not unravel the early events in PE development that precede abnormal placentation. We advocate that the combined use of different models is still required to enable novel developments regarding PE pathogenesis and treatment. However, as the advances made in this field of research continue to grow, the refinement of these models will undoubtedly occur, leading to the discovery of new aspects of this disorder.

## References

[1] Kassebaum NJ, Barber RM, Bhutta ZA, Dandona L, Gething PW, Hay SI (2016). Global, regional, and national levels of maternal mortality, 1990–2015: a systematic analysis for the Global Burden of Disease Study 2015. The Lancet.

[2] Wang H, Bhutta ZA, Coates MM, Coggeshall M, Dandona L, Diallo K (2016). Global, regional, national, and selected subnational levels of stillbirths, neonatal, infant, and under-5 mortality, 1980–2015: a systematic analysis for the Global Burden of Disease Study 2015. The Lancet.

[3] Lowe SA, Bowyer L, Lust K, McMahon LP, Morton M, North RA (2015). SOMANZ guidelines for the management of hypertensive disorders of pregnancy 2014. Aust N Z J Obstet Gynaecol.

[4] Magee LA, Nicolaides KH, Von Dadelszen P (2022). Preeclampsia. N Engl J Med.

[5] Ma’ayeh M, Rood KM, Kniss D, Costantine MM. Novel interventions for the prevention of preeclampsia. Curr Hypertens Rep. 2020;22(2):1–8. 10.1007/s11906-020-1026-8 PMC8237364 32052203

[6] Sones JL, Cha J, Woods AK, Bartos A, Heyward CY, Lob HE, et al. Decidual Cox2 inhibition improves fetal and maternal outcomes in a preeclampsia-like mouse model. JCI insight. 2016;1(3). 10.1172/jci.insight.75351 PMC4855694 27159542

[7] Roberts JM, editor Pathophysiology of ischemic placental disease. Semin Perinatol; 2014: Elsevier. 10.1053/j.semperi.2014.03.005 PMC4040272 24836825

[8] Thadhani R, Mutter WP, Wolf M, Levine RJ, Taylor RN, Sukhatme VP (2004). First trimester placental growth factor and soluble fms-like tyrosine kinase 1 and risk for preeclampsia. J Clin Endocrinol Metab.

[9] Powe CE, Levine RJ, Karumanchi SA (2011). Preeclampsia, a disease of the maternal endothelium: the role of antiangiogenic factors and implications for later cardiovascular disease. Circulation.

[10] Brown MA, Magee LA, Kenny LC, Karumanchi SA, McCarthy FP, Saito S (2018). Hypertensive disorders of pregnancy: ISSHP classification, diagnosis, and management recommendations for international practice. Hypertension.

[11] Aneman I, Pienaar D, Suvakov S, Simic TP, Garovic VD, McClements L (2020). Mechanisms of key innate immune cells in early- and late-onset preeclampsia. Front Immunol.

[12] Huppertz B (2018). The critical role of abnormal trophoblast development in the etiology of preeclampsia. Curr Pharm Biotechnol.

[13] Phipps E, Prasanna D, Brima W, Jim B (2016). Preeclampsia: updates in pathogenesis, definitions, and guidelines. Clin J Am Soc Nephrol.

[14] Rolnik DL, Wright D, Poon LC, O'Gorman N, Syngelaki A, de Paco MC (2017). Aspirin versus placebo in pregnancies at high risk for preterm preeclampsia. N Engl J Med.

[15] Bujold E, Roberge S, Lacasse Y, Bureau M, Audibert F, Marcoux S (2010). Prevention of preeclampsia and intrauterine growth restriction with aspirin started in early pregnancy: a meta-analysis. Obstet Gynecol.

[16] Mosca L, Benjamin EJ, Berra K, Bezanson JL, Dolor RJ, Lloyd-Jones DM (2011). Effectiveness-based guidelines for the prevention of cardiovascular disease in women—2011 update: a guideline from the American Heart Association. Circulation.

[17] Bushnell C, McCullough LD, Awad IA, Chireau MV, Fedder WN, Furie KL (2014). Guidelines for the prevention of stroke in women: a statement for healthcare professionals from the American Heart Association/American Stroke Association. Stroke.

[18] Gatford KL, Andraweera PH, Roberts CT, Care AS (2020). Animal models of preeclampsia: causes, consequences, and interventions. Hypertension.

[19] Alexander BT, Kassab SE, Miller MT, Abram SR, Reckelhoff JF, Bennett WA (2001). Reduced uterine perfusion pressure during pregnancy in the rat is associated with increases in arterial pressure and changes in renal nitric oxide. Hypertension.

[20] Khalil RA, Granger JP (2002). Vascular mechanisms of increased arterial pressure in preeclampsia: lessons from animal models. American Journal of Physiology-Regulatory, Integrative and Comparative Physiology.

[21] LaMarca B, Amaral LM, Harmon AC, Cornelius DC, Faulkner JL, Cunningham MW (2016). Placental ischemia and resultant phenotype in animal models of preeclampsia. Curr Hypertens Rep.

[22] Ramesar S, Mackraj I, Gathiram P, Moodley J (2011). Sildenafil citrate decreases sFlt-1 and sEng in pregnant L-NAME treated Sprague-Dawley rats. European Journal of Obstetrics & Gynecology and Reproductive Biology.

[23] Santillan MK, Santillan DA, Scroggins SM, Min JY, Sandgren JA, Pearson NA (2014). Vasopressin in preeclampsia: a novel very early human pregnancy biomarker and clinically relevant mouse model. Hypertension.

[24] Doridot L, Passet B, Méhats C, Barbaux S, Mondon F, Vilotte M (2012). STOX1 overexpression in mice induces severe preeclampsia-like symptoms prevented by aspirin at low doses. J Reprod Immunol.

[25] Maynard SE, Min J-Y, Merchan J, Lim K-H, Li J, Mondal S (2003). Excess placental soluble fms-like tyrosine kinase 1 (sFlt1) may contribute to endothelial dysfunction, hypertension, and proteinuria in preeclampsia. J Clin Investig.

[26] Santillan MK, Pelham CJ, Ketsawatsomkron P, Santillan DA, Davis DR, Devor EJ (2015). Pregnant mice lacking indoleamine 2, 3-dioxygenase exhibit preeclampsia phenotypes. Physiol Rep.

[27] Nishizawa H, Hasegawa K, Suzuki M, Achiwa Y, Kato T, Saito K (2008). Mouse model for allogeneic immune reaction against fetus recapitulates human pre-eclampsia. J Obstet Gynaecol Res.

[28] Gillis EE, Williams JM, Garrett MR, Mooney JN, Sasser JM (2015). The Dahl salt-sensitive rat is a spontaneous model of superimposed preeclampsia. American Journal of Physiology-Regulatory, Integrative and Comparative Physiology.

[29] Davisson RL, Hoffmann DS, Butz GM, Aldape G, Schlager G, Merrill DC (2002). Discovery of a spontaneous genetic mouse model of preeclampsia. Hypertension.

[30] Cushen SC, Goulopoulou S (2017). New models of pregnancy-associated hypertension. Am J Hypertens.

[31] •• Bakrania BA, George EM, Granger JP. Animal models of preeclampsia: investigating pathophysiology and therapeutic targets. Am J Obstet Gynecol. 2021. **This article provides a comprehensive review of the most widely studied and novel animal models created to study PE.** 10.1016/j.ajog.2020.10.025 PMC8141071 33722383

[32] Soobryan N, Murugesan S, Phoswa W, Gathiram P, Moodley J, Mackraj I (2017). The effects of sildenafil citrate on uterine angiogenic status and serum inflammatory markers in an L-NAME rat model of pre-eclampsia. Eur J Pharmacol.

[33] Doridot L, Delpoux A, Lucas B, Vaiman D (2013). Preeclampsia-like syndrome in STOX1 overexpressing mice: defects in immune regulation?: P-134. Am J Reprod Immunol.

[34] Ho L, Van Dijk M, Chye STJ, Messerschmidt DM, Chng SC, Ong S (2017). ELABELA deficiency promotes preeclampsia and cardiovascular malformations in mice. Science.

[35] Makris A, Thornton C, Thompson J, Thomson S, Martin R, Ogle R (2007). Uteroplacental ischemia results in proteinuric hypertension and elevated sFLT-1. Kidney Int.

[36] Baijnath S, Soobryan N, Mackraj I, Gathiram P, Moodley J (2014). The optimization of a chronic nitric oxide synthase (NOS) inhibition model of pre-eclampsia by evaluating physiological changes. European Journal of Obstetrics & Gynecology and Reproductive Biology.

[37] NICE. Pre-eclampsia. Hypertens Pregnancy [Internet]. 2021 30 October 2021:[1–21 pp.]. Available from: http://pathways.nice.org.uk/pathways/hypertension-in-pregnancy.

[38] Salahuddin S, Wenger JB, Zhang D, Thadhani R, Karumanchi SA, Rana S (2016). KRYPTOR-automated angiogenic factor assays and risk of preeclampsia-related adverse outcomes. Hypertens Pregnancy.

[39] Andersen LLT, Helt A, Sperling L, Overgaard M (2021). Decision threshold for Kryptor sFlt-1/PlGF ratio in women with suspected preeclampsia: retrospective study in a routine clinical setting. J Am Heart Assoc.

[40] Clark DA (2014). The use and misuse of animal analog models of human pregnancy disorders. J Reprod Immunol.

[41] El-Hashash AH, Warburton D, Kimber SJ (2010). Genes and signals regulating murine trophoblast cell development. Mech Dev.

[42] Maltepe E, Bakardjiev AI, Fisher SJ (2010). The placenta: transcriptional, epigenetic, and physiological integration during development. J Clin Investig.

[43] Coan P, Conroy N, Burton G, Ferguson-Smith A (2006). Origin and characteristics of glycogen cells in the developing murine placenta. Developmental dynamics: an official publication of the American Association of Anatomists.

[44] Pijnenborg R, Vercruysse L. Animal models of deep trophoblast invasion. In: Pijnenborg R, Brosens I, Romero R, editors. Placental bed disorders Cambridge: Cambridge University Press; 2010. p. 127e39.

[45] Soares MJ, Varberg KM, Iqbal K (2018). Hemochorial placentation: development, function, and adaptations. Biol Reprod.

[46] Wong AY, Kulandavelu S, Whiteley KJ, Qu D, Langille BL, Adamson SL (2002). Maternal cardiovascular changes during pregnancy and postpartum in mice. American Journal of Physiology-Heart and Circulatory Physiology.

[47] Kulandavelu S, Qu D, Adamson SL (2006). Cardiovascular function in mice during normal pregnancy and in the absence of endothelial NO synthase. Hypertension.

[48] Maltepe E, Fisher SJ (2015). Placenta: the forgotten organ. Annu Rev Cell Dev Biol.

[49] Murphy SR, LaMarca BBD, Cockrell K, Granger JP (2010). Role of endothelin in mediating soluble fms-like tyrosine kinase 1–induced hypertension in pregnant rats. Hypertension.

[50] LaMarca B, Parrish M, Ray LF, Murphy SR, Roberts L, Glover P (2009). Hypertension in response to autoantibodies to the angiotensin II type I receptor (AT1-AA) in pregnant rats: role of endothelin-1. Hypertension.

[51] LaMarca BBD, Cockrell K, Sullivan E, Bennett W, Granger JP (2005). Role of endothelin in mediating tumor necrosis factor-induced hypertension in pregnant rats. Hypertension.

[52] Faulkner JL, Cornelius DC, Amaral LM, Harmon AC, Cunningham MW, Darby MM (2016). Vitamin D supplementation improves pathophysiology in a rat model of preeclampsia. American Journal of Physiology-Regulatory, Integrative and Comparative Physiology.

[53] Tian X, Ma S, Wang Y, Hou L, Shi Y, Yao M (2016). Effects of placental ischemia are attenuated by 1, 25-dihydroxyvitamin D treatment and associated with reduced apoptosis and increased autophagy. DNA Cell Biol.

[54] Darby MM, Wallace K, Cornelius D, Chatman KT, Mosely JN, Martin JN, et al. Vitamin D supplementation suppresses hypoxia-stimulated placental cytokine secretion, hypertension and CD4+ T cell stimulation in response to placental ischemia. Medical journal of obstetrics and gynecology. 2013;1(2). PMC4235666 25414911

[55] Song J, Li Y, An R (2017). Vitamin D restores angiogenic balance and decreases tumor necrosis factor-α in a rat model of pre-eclampsia. J Obstet Gynaecol Res.

[56] Zenclussen AC, Fest S, Joachim R, Klapp BF, Arck PC (2004). Introducing a mouse model for pre-eclampsia: adoptive transfer of activated Th1 cells leads to pre-eclampsia-like symptoms exclusively in pregnant mice. Eur J Immunol.

[57] Novotny S, Wallace K, Herse F, Moseley J, Darby M, Heath J, et al. CD4+ T cells play a critical role in mediating hypertension in response to placental ischemia. J Hypertens. open access. 2013;2. 10.4172/2167-1095.1000116 PMC4231445 25401050

[58] Harmon A, Cornelius D, Amaral L, Paige A, Herse F, Ibrahim T (2015). IL-10 supplementation increases Tregs and decreases hypertension in the RUPP rat model of preeclampsia. Hypertens Pregnancy.

[59] Wallace K, Richards S, Dhillon P, Weimer A, Edholm E-s, Bengten E, et al. CD4+ T-helper cells stimulated in response to placental ischemia mediate hypertension during pregnancy. Hypertension. 2011;57(5):949–55. 10.1161/HYPERTENSIONAHA.110.168344 PMC3144629 21464392

[60] Bauer AJ, Banek CT, Needham K, Gillham H, Capoccia S, Regal JF (2013). Pravastatin attenuates hypertension, oxidative stress, and angiogenic imbalance in rat model of placental ischemia-induced hypertension. Hypertension.

[61] Ahmed A, Williams DJ, Cheed V (2020). Pravastatin for early-onset pre-eclampsia: a randomised, blinded, placebo-controlled trial. BJOG.

[62] Costantine MM, West H, Wisner KL, Caritis S, Clark S, Venkataramanan R, et al. A randomized pilot clinical trial of pravastatin versus placebo in pregnant patients at high-risk of preeclampsia. Am J Obstet Gynecol. 2021. 10.1016/j.ajog.2021.05.018 PMC8611118 34033812

[63] Spradley FT, Tan AY, Joo WS, Daniels G, Kussie P, Karumanchi SA (2016). Placental growth factor administration abolishes placental ischemia-induced hypertension. Hypertension.

[64] Dokras A, Hoffmann DS, Eastvold JS, Kienzle MF, Gruman LM, Kirby PA (2006). Severe feto-placental abnormalities precede the onset of hypertension and proteinuria in a mouse model of preeclampsia. Biol Reprod.

[65] McCarthy F, Kingdom J, Kenny L, Walsh S (2011). Animal models of preeclampsia; uses and limitations. Placenta.

[66] Woods AK, Hoffmann DS, Weydert CJ, Butler SD, Zhou Y, Sharma RV (2011). Adenoviral delivery of VEGF121 early in pregnancy prevents spontaneous development of preeclampsia in BPH/5 mice. Hypertension.

[67] Thadhani R, Kisner T, Hagmann H, Bossung V, Noack S, Schaarschmidt W (2011). Pilot study of extracorporeal removal of soluble fms-like tyrosine kinase 1 in preeclampsia. Circulation.

[68] Thadhani R, Hagmann H, Schaarschmidt W, Roth B, Cingoez T, Karumanchi SA (2016). Removal of soluble fms-like tyrosine kinase-1 by dextran sulfate apheresis in preeclampsia. J Am Soc Nephrol.

[69] NICE. Antenatal care: routine care for the healthy pregnant woman. Clinical Guideline CG62. London, NICE, 2008. 2008.

[70] Bytautiene E, Lu F, Tamayo EH, Hankins GD, Longo M, Kublickiene K (2010). Long-term maternal cardiovascular function in a mouse model of sFlt-1-induced preeclampsia. American Journal of Physiology-Heart and Circulatory Physiology.

[71] Alpoim PN, Godoi LC, Freitas LG, Gomes KB, Dusse LM. Assessment of L-arginine asymmetric 1 dimethyl (ADMA) in early-onset and late-onset (severe) preeclampsia. Nitric Oxide. 2013;33:81–2. 10.1016/j.niox.2013.07.006 23876347

[72] Alpoim PN, Gomes KB, de Barros Pinheiro M, Godoi LC, Jardim LL, Muniz LG, et al. Polymorphisms in endothelial nitric oxide synthase gene in early and late severe preeclampsia. Nitric Oxide. 2014;42:19–23. 10.1016/j.niox.2014.07.006 25106888

[73] Molnär M, Söto T, Tóth T, Hertelendy F (1994). Prolonged blockade of nitric oxide synthesis in gravid rats produces sustained hypertension, proteinuria, thrombocytopenia, and intrauterine growth retardation. Am J Obstet Gynecol.

[74] Motta C, Grosso C, Zanuzzi C, Molinero D, Picco N, Bellingeri R (2015). Effect of sildenafil on pre-eclampsia-like mouse model induced by L-name. Reprod Domest Anim.

[75] Salas SaP, Altermatt F, Campos M, Giacaman A, Rosso P. Effects of long-term nitric oxide synthesis inhibition on plasma volume expansion and fetal growth in the pregnant rat. Hypertension. 1995;26(6):1019–23. 10.1161/01.hyp.26.6.1019 7498960

[76] Ma R, Sun M, Yang Z (2010). Effects of preeclampsia-like symptoms at early gestational stage on feto-placental outcomes in a mouse model. Chin Med J.

[77] Ramesar S, Mackraj I, Gathiram P, Moodley J (2010). Sildenafil citrate improves fetal outcomes in pregnant, l-NAME treated, Sprague-Dawley rats. European Journal of Obstetrics & Gynecology and Reproductive Biology.

[78] Rossoni G, Manfredi B, De Gennaro CV, Berti M, Guazzi M, Berti F (2007). Sildenafil reduces L-NAME-induced severe hypertension and worsening of myocardial ischaemia–reperfusion damage in the rat. Br J Pharmacol.

[79] Baijnath S, Murugesan S, Mackraj I, Gathiram P, Moodley J (2017). The effects of sildenafil citrate on urinary podocin and nephrin mRNA expression in an L-NAME model of pre-eclampsia. Mol Cell Biochem.

[80] Samangaya RA, Mires G, Shennan A, Skillern L, Howe D, McLeod A (2009). A randomised, double-blinded, placebo-controlled study of the phosphodiesterase type 5 inhibitor sildenafil for the treatment of preeclampsia. Hypertens Pregnancy.

[81] Nassar AH, Masrouha KZ, Itani H, Abi Nader K, Usta IM (2012). Effects of sildenafil in Nω-nitro-L-arginine methyl ester–induced intrauterine growth restriction in a rat model. Am J Perinatol.

[82] Erlandsson L, Nääv Å, Hennessy A, Vaiman D, Gram M, Åkerström B (2016). Inventory of novel animal models addressing etiology of preeclampsia in the development of new therapeutic/intervention opportunities. Am J Reprod Immunol.

[83] • Waker CA, Kaufman MR, Brown TL. Current state of preeclampsia mouse models: approaches, relevance, and standardization. Front Physiol. 2021;12. **This article reviews and summarizes the key findings from current and novel murine models of PE.** 10.3389/fphys.2021.681632 PMC8284253 34276401

[84] Huang PL, Huang Z, Mashimo H, Bloch KD, Moskowitz MA, Bevan JA (1995). Hypertension in mice lacking the gene for endothelial nitric oxide synthase. Nature.

[85] Shesely EG, Gilbert C, Granderson G, Carretero CD, Carretero OA, Beierwaltes WH (2001). Nitric oxide synthase gene knockout mice do not become hypertensive during pregnancy. Am J Obstet Gynecol.

[86] Yadav VR, Teng B, Mustafa SJ (2019). Enhanced A(1) adenosine receptor-induced vascular contractions in mesenteric artery and aorta of in L-NAME mouse model of hypertension. Eur J Pharmacol.

[87] Kalliolias GD, Ivashkiv LB (2016). TNF biology, pathogenic mechanisms and emerging therapeutic strategies. Nat Rev Rheumatol.

[88] Hayashi M, Ueda Y, Yamaguchi T, Sohma R, Shibazaki M, Ohkura T (2005). Tumor necrosis factor-α in the placenta is not elevated in pre-eclamptic patients despite its elevation in peripheral blood. Am J Reprod Immunol.

[89] Cackovic M, Buhimschi CS, Zhao G, Funai EF, Norwitz ER, Kuczynski E (2008). Fractional excretion of tumor necrosis factor-alpha in women with severe preeclampsia. Obstet Gynecol.

[90] Founds SA, Powers RW, Patrick TE, Ren D, Harger GF, Markovic N (2008). A comparison of circulating TNF-α in obese and lean women with and without preeclampsia. Hypertens Pregnancy.

[91] Tosun M, Celik H, Avci B, Yavuz E, Alper T, Malatyalioğlu E (2010). Maternal and umbilical serum levels of interleukin-6, interleukin-8, and tumor necrosis factor-α in normal pregnancies and in pregnancies complicated by preeclampsia. J Matern Fetal Neonatal Med.

[92] Peraçoli JC, Bannwart-Castro CF, Romao M, Weel IC, Ribeiro VR, Borges VT (2013). High levels of heat shock protein 70 are associated with pro-inflammatory cytokines and may differentiate early-from late-onset preeclampsia. J Reprod Immunol.

[93] Alexander BT, Cockrell KL, Massey MB, Bennett WA, Granger JP (2002). Tumor necrosis factor–α–induced hypertension in pregnant rats results in decreased renal neuronal nitric oxide synthase expression. Am J Hypertens.

[94] LaMarca B, Wallukat G, Llinas M, Herse F, Dechend R, Granger JP (2008). Autoantibodies to the angiotensin type I receptor in response to placental ischemia and tumor necrosis factor α in pregnant rats. Hypertension.

[95] Selvan S, Dowling J, Kelly W, Lin J (2016). Indoleamine 2, 3-dioxygenase (IDO): biology and target in cancer immunotherapies. Curr Cancer Drug Targets.

[96] Kudo Y, Boyd C, Sargent I, Redman C (2000). Modulation of indoleamine 2, 3-dioxygenase by interferon-γ in human placental chorionic villi. Mol Hum Reprod.

[97] Kudo Y, Boyd C, Sargent IL, Redman CW (2003). Decreased tryptophan catabolism by placental indoleamine 2, 3-dioxygenase in preeclampsia. Am J Obstet Gynecol.

[98] Santoso DIS, Rogers P, Wallace EM, Manuelpillai U, Walker D, Subakir SB (2002). Localization of indoleamine 2, 3-dioxygenase and 4-hydroxynonenal in normal and pre-eclamptic placentae. Placenta.

[99] Nishizawa H, Hasegawa K, Suzuki M, Kamoshida S, Kato T, Saito K (2007). The etiological role of allogeneic fetal rejection in pre-eclampsia. Am J Reprod Immunol.

[100] Iwahashi N, Yamamoto M, Nanjo S, Toujima S, Minami S, Ino K (2017). Downregulation of indoleamine 2, 3-dioxygenase expression in the villous stromal endothelial cells of placentas with preeclampsia. J Reprod Immunol.

[101] Zhou CC, Zhang Y, Irani RA, Zhang H, Mi T, Popek EJ (2008). Angiotensin receptor agonistic autoantibodies induce pre-eclampsia in pregnant mice. Nat Med.

[102] Campbell N, LaMarca B, Cunningham MW (2018). The role of agonistic autoantibodies to the angiotensin II type 1 receptor (AT1-AA) in pathophysiology of preeclampsia. Curr Pharm Biotechnol.

[103] Marshall SA, Hannan NJ, Jelinic M, Nguyen TP, Girling JE, Parry LJ (2018). Animal models of preeclampsia: translational failings and why. American Journal of Physiology-Regulatory, Integrative and Comparative Physiology.

[104] LaMarca B, Parrish MR, Wallace K (2012). Agonistic autoantibodies to the angiotensin II type I receptor cause pathophysiologic characteristics of preeclampsia. Gend Med.

[105] Tinsley JH, Chiasson VL, Mahajan A, Young KJ, Mitchell BM (2009). Toll-like receptor 3 activation during pregnancy elicits preeclampsia-like symptoms in rats. Am J Hypertens.

[106] Goulopoulou S, McCarthy CG, Webb RC (2016). Toll-like receptors in the vascular system: sensing the dangers within. Pharmacol Rev.

[107] Koga K, Izumi G, Mor G, Fujii T, Osuga Y (2014). Toll-like receptors at the maternal-fetal interface in normal pregnancy and pregnancy complications. Am J Reprod Immunol.

[108] Chatterjee P, Chiasson VL, Kopriva SE, Young KJ, Chatterjee V, Jones KA (2011). Interleukin 10 deficiency exacerbates Toll-like receptor 3–induced preeclampsia-like symptoms in mice. Hypertension.

[109] Chatterjee P, Weaver LE, Doersch KM, Kopriva SE, Chiasson VL, Allen SJ, et al. Placental Toll-like receptor 3 and Toll-like receptor 7/8 activation contributes to preeclampsia in humans and mice. 2012. 10.1371/journal.pone.0041884 PMC3407075 22848646

[110] Goulopoulou S, Wenceslau CF, McCarthy CG, Matsumoto T, Webb RC (2016). Exposure to stimulatory CpG oligonucleotides during gestation induces maternal hypertension and excess vasoconstriction in pregnant rats. American Journal of Physiology-Heart and Circulatory Physiology.

[111] Thaxton JE, Romero R, Sharma S (2009). TLR9 activation coupled to IL-10 deficiency induces adverse pregnancy outcomes. J Immunol.

[112] Scharfe-Nugent A, Corr SC, Carpenter SB, Keogh L, Doyle B, Martin C (2012). TLR9 provokes inflammation in response to fetal DNA: mechanism for fetal loss in preterm birth and preeclampsia. J Immunol.

[113] Prater MR, Johnson VJ, Germolec DR, Luster MI, Holladay SD (2006). Maternal treatment with a high dose of CpG ODN during gestation alters fetal craniofacial and distal limb development in C57BL/6 mice. Vaccine.

[114] van Dijk M, Drewlo S, Oudejans CB (2010). Differential methylation of STOX1 in human placenta. Epigenetics.

[115] Van Dijk M, van Bezu J, van Abel D, Dunk C, Blankenstein MA, Oudejans CB (2010). The STOX1 genotype associated with pre-eclampsia leads to a reduction of trophoblast invasion by α-T-catenin upregulation. Hum Mol Genet.

[116] Haram K, Mortensen JH, Nagy B. Genetic aspects of preeclampsia and the HELLP syndrome. J pregnancy. 2014;2014. 10.1155/2014/910751 PMC4060423 24991435

[117] George EM, Bidwell GL. STOX1: a new player in preeclampsia? : Am Heart Assoc; 2013. 10.1161/HYPERTENSIONAHA.111.00721 PMC4199576 23357180

[118] Collinot H, Marchiol C, Lagoutte I, Lager F, Siauve N, Autret G (2018). Preeclampsia induced by STOX1 overexpression in mice induces intrauterine growth restriction, abnormal ultrasonography and BOLD MRI signatures. J Hypertens.

[119] Ducat A, Doridot L, Calicchio R, Méhats C, Vilotte J-L, Castille J (2016). Endothelial cell dysfunction and cardiac hypertrophy in the STOX1 model of preeclampsia. Sci Rep.

[120] Miralles F, Collinot H, Boumerdassi Y, Ducat A, Duché A, Renault G (2019). Long-term cardiovascular disorders in the STOX1 mouse model of preeclampsia. Sci Rep.

[121] Gillis EE, Mooney JN, Garrett MR, Granger JP, Sasser JM (2016). Sildenafil treatment ameliorates the maternal syndrome of preeclampsia and rescues fetal growth in the dahl salt–sensitive rat. Hypertension.

[122] Sandgren JA, Deng G, Linggonegoro DW, Scroggins SM, Perschbacher KJ, Nair AR, et al. Arginine vasopressin infusion is sufficient to model clinical features of preeclampsia in mice. JCI insight. 2018;3(19). 10.1172/jci.insight.99403 PMC6237463 30282823

[123] Koshimizu T-a, Nakamura K, Egashira N, Hiroyama M, Nonoguchi H, Tanoue A. Vasopressin V1a and V1b receptors: from molecules to physiological systems. Physiol Rev. 2012;92(4):1813–64. 10.1152/physrev.00035.2011 23073632

[124] Buchwalter A, Van Dort C, Schultz S, Smith R, Le I, Abbott J (2008). Expression of VACM-1/cul5 mutant in endothelial cells induces MAPK phosphorylation and maspin degradation and converts cells to the angiogenic phenotype. Microvasc Res.

[125] Fuchs A, Fields M, Freidman S, Shemesh M, Ivell R. Oxytocin and the timing of parturition. Influence of oxytocin receptor gene expression, oxytocin secretion, and oxytocin-induced prostaglandin F2 alpha and E2 release. Adv Exp Med Biol. 1995;395:405–20. 8713995

[126] Scroggins SM, Santillan DA, Lund JM, Sandgren JA, Krotz LK, Hamilton WS (2018). Elevated vasopressin in pregnant mice induces T-helper subset alterations consistent with human preeclampsia. Clin Sci.

[127] Sones JL, Davisson RL (2016). Preeclampsia, of mice and women. Physiol Genomics.

[128] Ramdin S, Naicker T, Pillay V, Singh SD, Baijnath S, Mkhwanazi BN (2022). Physiological characterization of an arginine vasopressin rat model of preeclampsia. Syst Biol Reprod Med.

[129] Govender N, Ramdin S, Reddy R, Naicker T (2021). Transforming growth factor-beta and liver injury in an arginine vasopressin-induced pregnant rat model. Clin Exp Reprod Med.

[130] Care AS, Bourque SL, Morton JS, Hjartarson EP, Robertson SA, Davidge ST (2018). Reduction in regulatory T cells in early pregnancy causes uterine artery dysfunction in mice. Hypertension.

